# Effect of Anesthesia on Endoscopic Sinus Surgery Hemostasis: A State-of-the-Art Review

**DOI:** 10.7759/cureus.42467

**Published:** 2023-07-26

**Authors:** André De Sousa Machado

**Affiliations:** 1 ENT, Centro Hospitalar Universitário do Porto, Porto, PRT; 2 Medical Education, Faculdade Ciencias da Saude, Universidade da Beira Interior, Covilha, PRT

**Keywords:** functional endoscopic sinus surgery (fess), endoscopic sinus surgery (ess), otolaryngology, bleeding risk, total intravenous anesthesia (tiva)

## Abstract

Endoscopic sinus surgery (ESS) is the gold standard for the management of chronic rhinosinusitis, nasal polyposis, and other pathology involving paranasal sinus as tumors. Intraoperative bleeding during ESS can be challenging due to the narrow sinonasal surgical field, single working hand, and the use of endoscopic instruments, which may affect hemostasis. There is a role for the type of anesthesia technique used for intraoperative bleeding control. Total intravenous anesthesia (TIVA) and inhalational anesthesia (IA) are some of the techniques available for anesthetic purposes. While both techniques have their advantages and disadvantages, there is a need to compare their efficacy and safety to determine which technique is more appropriate for ESS. In this review, our main focus was to summarize the current evidence about the different techniques of anesthesia used during ESS. A systematic review of the PubMed/MEDLINE database was performed using specific English terms related to TIVA and IA/volatile anesthesia used during ESS. A total of 548 publications were considered. Among these, 329 studies did not fulfill the criteria for inclusion in the systematic review, resulting in the inclusion of only 132 publications: 13 systematic reviews, 32 reviews, 92 randomized controlled trials, and 13 meta-analyses. The state of the art favors the use of TIVA during ESS due to its significant improvement in the intraoperative surgical field with less blood loss. Further studies aim to compare long-term nasal status with objective tools, ideally in similar pathology with the same surgeon.

## Introduction and background

Endoscopic sinus surgery (ESS) is a surgical procedure that is the gold standard for the surgical management of chronic rhinosinusitis (CRS), nasal polyposis, and other pathology affecting paranasal sinus, such as tumoral pathology. Sometimes, there is a need to combine this procedure with other neurosurgical techniques (such as the transethmoid‐paraseptal approach) in order to reach the central skull base. It is essential for ESS to achieve optimal intraoperative surgical field visibility and minimize blood loss, which is one of the critical factors for successful outcomes. Factors that can influence bleeding during ESS include the use of antiplatelet and/or anticoagulant drugs, hypertension, the Lund-Mackay CT score, damage to vessels due to the altered anatomy, lack of surgeon experience, extensive sinus disease, revision surgery, and poor visualization [1-5]. Intraoperative bleeding during ESS can be challenging due to the narrow sinonasal surgical field, single working hand, and the use of endoscopic instruments, which may affect hemostasis [3]. Also, there is a role for the type of anesthesia used for intraoperative bleeding control. It is worth mentioning that there are pharmacological and non-pharmacological measures and that the use of permissive hypotension during anesthesia can help both techniques achieve optimal field conditions. Total intravenous anesthesia (TIVA) and inhalational anesthesia (IA) are the most used anesthetic techniques during ESS. While both techniques have their advantages and disadvantages, there is a need to compare their efficacy and safety to determine which technique is more appropriate for ESS, bearing in mind that it is a field of crescent interest by both surgeons and anesthetists and considering the patient’s perioperative safety and comfort. In this present state-of-the-art review, our main focus was to summarize the current evidence about the different types of anesthesia used during ESS and their differences for the surgeon based on intraoperative bleeding.

## Review

**Figure 1:**
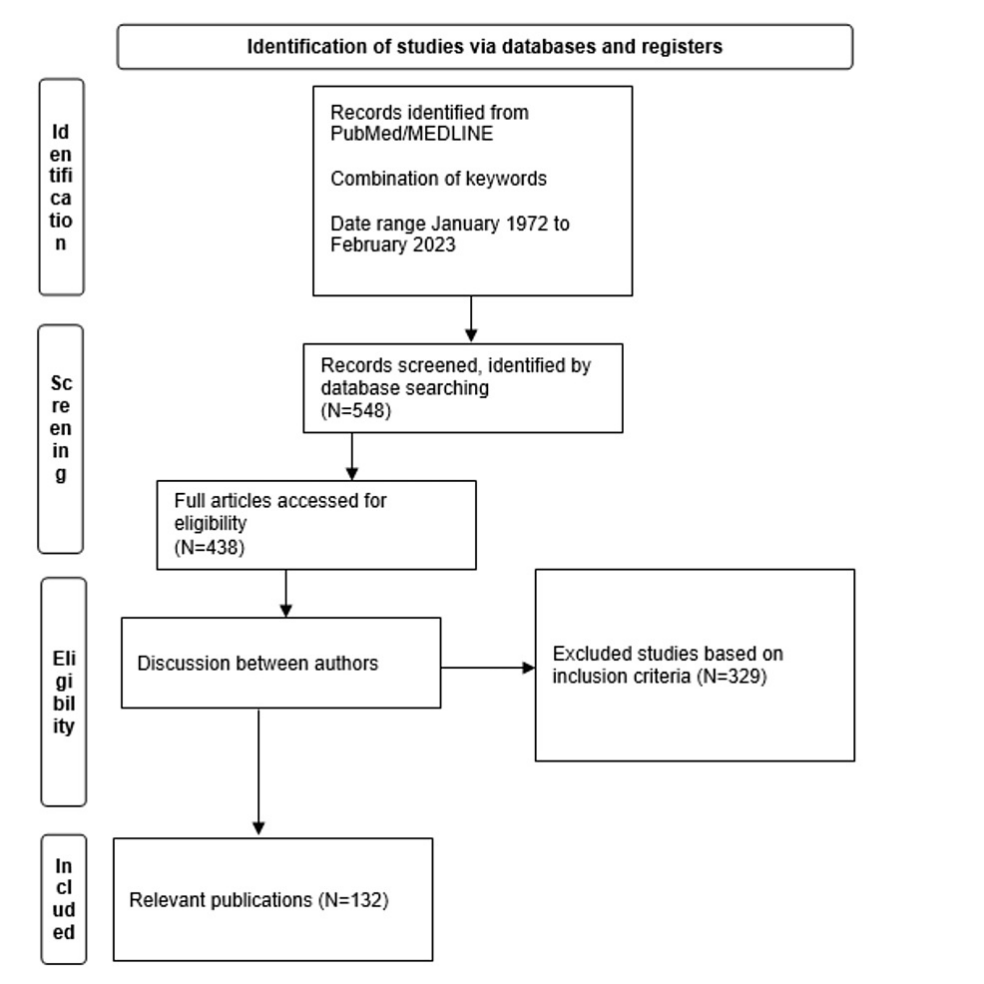
A PRISMA flowchart

A systematic review of the PubMed/MEDLINE database was performed using specific English terms related to TIVA and IA used during ESS. The terms used were “endoscopic sinus surgery,” “anesthesia,” “total intravenous anesthesia,” “volatile anesthesia,” and “inhalational anesthesia.” It included prospective and retrospective studies, experimental research, meta-analyses, and systematic reviews. Our focus was on publications that compared TIVA and IA for ESS in adults. The articles were selected based on their relevance to the role of anesthesia in the intraoperative field and long-term nasal outcomes. We analyzed the selected articles critically and summarized their implications for practice. The PRISMA criteria were used to select the articles, as shown in Figure 1. Ethics committee approval was not required.

From 1972 onward, a total of 548 publications were detected. Among these, 329 studies did not fulfill the criteria for inclusion in the systematic review, resulting in the inclusion of only 132 publications: 13 systematic reviews, 32 reviews, 92 randomized controlled trials, and 13 meta-analyses.

Role of anesthesia in the intraoperative field

ESS aims to create a clear surgical field during the procedure by minimizing bleeding and avoiding injuries to the vascular, cerebrospinal fluid, and orbital tissues [1,6-8]. To achieve this, various techniques are used to reduce blood flow to the intraoperative field. One technique used in ESS is the sphenopalatine artery block. This block involves injecting a local anesthetic with adrenaline through the greater palatine canal to block the sphenopalatine artery, which is the main feeding vessel to the lateral wall and most of the septum. This technique is believed to reduce the amount of bleeding and improve the surgical field during ESS [9]. Another technique used to reduce bleeding is the use of a combination of medications, such as tranexamic acid [10]. Tranexamic acid is a safe, cheap, and effective medication that can be routinely administered when the anticipated blood loss is high. It is a synthetic antifibrinolytic medication that has shown promising results in reducing blood loss and improving the outcomes of various surgical nasal procedures, such as ESS, rhinoplasty, and septorhinoplasty [11]. The drug has also been used as a lifesaving antifibrinolytic agent in plastic and reconstructive surgery to reduce intraoperative blood loss [12]. In ESS, the use of tranexamic acid has been shown to improve the outcome and reduce bleeding, which is one of the most devastating complications during the procedure [13]. Blood conservation techniques are also used in ESS to reduce the risk of bleeding and to improve patient outcomes [14]. These techniques include preoperative optimization of hemoglobin, management of comorbidities and medications, intraoperative blood preservation techniques, and careful postoperative management. Surgical blood conservation techniques used during ESS include preoperative autologous donation and intraoperative hemodilution during surgery [15]. Multiple techniques are used to reduce blood flow to the intraoperative field during functional ESS (FESS). One study investigated whether the patient’s position during surgery (15° reverse Trendelenburg position vs horizontal position) had an impact on the endoscopic field of view and intraoperative blood loss, with the results still being uncertain [16]. TIVA and IA are two options for ESS. TIVA is characterized by the use of hypnotic agents, such as propofol in combination with opioids, while IA involves the use of volatile agents. The main advantage of TIVA is the reduced incidence of postoperative nausea and vomiting, faster emergence from anesthesia, and reduced emergence of delirium in pediatric patients and cognitive impairment in the elderly. The costs of TIVA are usually higher than those of IA, but these differences are difficult to measure due to various factors. The choice between TIVA and IA should be based on individual patient needs, surgeon preferences, and the anesthesia provider’s expertise [17,18]. TIVA is commonly used for ESS because it provides the surgeon with a stable and controlled environment for the procedure, reducing the risk of complications that arise from uncontrolled bleeding such as iatrogenic damage to nearby structures, such as the orbit and the brain, which can lead to a cerebral spinal fluid leak, meningitis, and brain abscess [19]. Additionally, TIVA does not have any long-term effects on the nasal outcomes of ESS, as the drugs are metabolized and eliminated from the body after the procedure [20,21].

TIVA, IA, and blood loss during ESS

During ESS, bleeding can compromise the surgeon’s intraoperative visibility, and both TIVA and IA are used to mitigate it. Studies comparing IA and TIVA during FESS have produced many incongruent results. One study in 2018 found that TIVA was associated with improved surgical fields and decreased bleeding during ESS when compared to IA [22]. The patients were enrolled in a double-blind, randomized controlled trial, where IA or TIVA were administered randomly to subjects during ESS [22]. The Wormald Surgical Field Grading Scale and Boezaart score were used by three blinded reviewers to grade intraoperative visibility as the main outcome. Both scores were lower in the TIVA arm. Also, it is worth mentioning that the groups were homogeneous in all compared baseline characteristics, and by doing so, the authors concluded that the use of TIVA versus IA resulted in a statistically significant improvement in the intraoperative surgical field during ESS with less blood loss. Less postoperative nausea, better intraoperative hemodynamic control, cost of anesthetic agents, and superior surgical field visibility were the main criteria that advocated the use of TIVA in ESS, according to the survey [22]. A survey published by Yoshiyasu et al., in 2018, concluded that TIVA may be beneficial for patients with CRS and high-grade inflammatory disease as it can reduce mucosal bleeding, which can cause difficult visualization during ESS [23]. Chaaban et al., in 2013, showed that TIVA resulted in better intraoperative conditions than IA in patients, with a high Lund-Mackay score (>12) without statistically significant differences in mean blood pressure and heart rate between groups [24]. Miłoński et al., in 2012, assessed the effect of three different types of anesthesia on perioperative bleeding control and analyzed the mean arterial blood pressure and heart rate in patients undergoing ESS [25]. Ninety adult patients were randomly assigned to one of three types/groups of general anesthesia. Mean anesthesia and surgery duration were inferior in the TIVA group. Mean blood loss and mean blood loss rate during surgery were also inferior in the TIVA group [25]. A meta-analysis by Kolia and Man, in 2019, showed that TIVA with propofol, compared to IA, can improve the surgical field quality and reduce blood loss, while lessening the operative time for ESS; remifentanil is the preferred opioid for TIVA during ESS [26]. There is an ongoing study that compares the effectiveness of TIVA to IA in terms of intraoperative hemodynamic parameters and recovery profile [27]. There is another study on track that compares TIVA to intravenous tranexamic acid, followed by general IA to determine which one provides a better surgical field viewing quality [28]. In conclusion, TIVA with propofol can improve the surgical field quality and reduce blood loss, while lessening the operative time for ESS, compared to IA (Table 1).

**Table 1:** Best strategy for better outcomes on blood loss in ESS ESS, endoscopic sinus surgery; TIVA, total intravenous anesthesia

	Best strategy for better outcomes on blood loss in ESS
Brunner et al., 2018 [22]	TIVA
Yoshiyasu et al., 2020 [23]	TIVA
Chaaban et al., 2013 [24]	TIVA
Milonski et al., 2013 [25]	TIVA
Kolia and Man, 2019 [26]	TIVA

Postoperative outcomes of ESS comparing TIVA and IA

Lu et al., in 2020, showed that patients who received TIVA in ESS had better intraoperative surgical conditions and had faster recovery compared to those who received IA [21]. Liu et al., in 2019, studied a cohort of patients undergoing ESS who received TIVA and had a better quality of recovery at six hours post-surgery compared to those who received IA, based on the quality of recovery-40 questionnaire (QoR-40) that reports the incidence of nausea and vomiting, blood loss, and pain [29]. The TIVA group also experienced less blood loss during the procedure, which is 150 mL versus 200 mL in the desflurane group. Moreover, the study revealed that patients with high Lund-Mackay scores, indicating severe sinusitis, had lower QoR-40 scores. Overall, the study suggests that TIVA may be a more favorable option than IA for ESS patients because it leads to better early recovery from anesthesia and reduced intraoperative blood loss [29]. In a randomized clinical trial published in 2022 by Kim et al., it was found that TIVA was more effective than IA in reducing emergence agitation and coughing, which was different from previous research. The evaluation in this study used graded scales, whereas previous studies did not. TIVA was associated with better antiemetic effects during the post-anesthesia care unit stay, which is a benefit of propofol-based TIVA compared to IA [30]. In a randomized clinical trial published in 2020 by Heller et al., patients who underwent TIVA had significantly decreased mean and median pain scores in the post-anesthesia care unit compared to those who underwent IA. In this study, there was no difference in the rate of postoperative nausea and vomiting and in time to discharge between the two groups [31]. Montes et al. published a study, in 2002, on a cohort of patients who underwent nasal surgery and were equally divided between IA and TIVA. The frequency of nausea and vomiting was low in both groups. Only two patients from the IA group required ondansetron IV for vomiting, while no patients from the TIVA group needed treatment for nausea or vomiting. Patients in the TIVA group achieved home readiness criteria significantly faster. As for postoperative pain, six patients from the TIVA group and eight from the IA group were treated for pain with morphine IV, with no statistically significant difference [32] (Table 2). 

**Table 2:** Best strategy for better postoperative outcomes in ESS ESS, endoscopic sinus surgery; TIVA, total intravenous anesthesia; IA, inhaled anesthesia

	Best strategy for better postoperative outcomes in ESS
Lu et al., 2020 [21]	TIVA
Liu et al., 2019 [29]	TIVA
Kim et al., 2022 [30]	TIVA
Heller et al., 2020 [31]	IA similar to TIVA
Montes et al., 2002 [32]	IA similar to TIVA

Also, descriptive data of studies considering preoperative anticoagulation, intraoperative procoagulant use (tranexamic acid), amount of blood loss or surgical view during the procedure, and surgical view during the procedure were considered and are listed in Table 3.

**Table 3:** Descriptive data of studies considered TIVA, total intravenous anesthesia; IA, inhalational anesthesia; NR, non-reported

	Preoperative anticoagulation	Intraoperative procoagulant use (transexamic acid)	Amount of blood loss or surgical view during the procedure (IA + TIVA/TIVA/IA) in mL	Surgical view during the procedure
Brunner et al., 2018 [22]	No	No	(-/200/300)	Wormald Surgical Field Grading Scale of 3.5 in TIVA and 4.1 in IA
Chaaban et al., 2013 [24]	No	No	(-/194.4/131)	NR
Milonski et al., 2013 [25]	No	No	(365/225/340)	NR
Lu et al., 2020 [21]	No	No	(-/100/120)	Boezaart score was lower in the TIVA group
Heller et al., 2020 [31]	No	No	(-72.5/74.5)	Boezaart scores were similar between groups

## Conclusions

The state of the art favors the use of TIVA during ESS due to its significant improvement in the intraoperative surgical field with less blood loss. Further studies that aim to compare long-term nasal status with objective tools, ideally in similar pathology with the same surgeon, should be considered in the future for more data on this topic. Both TIVA and IA have their pros and cons, and the decision should be made based on careful consideration of the patient’s individual needs and preferences. Further studies are needed to determine the best approach for ESS, taking into account the patient’s individual needs and preferences.
